# A multivariate retrospective analysis of high‐grade gliomas: Survival and prognostic factors

**DOI:** 10.1002/cam4.7456

**Published:** 2024-08-09

**Authors:** Weiyan Shi, Xuanzhong Wang, Shiyu Liu, Zhuangzhuang Zheng, Lihua Dong, Xin Jiang

**Affiliations:** ^1^ Jilin Provincial Key Laboratory of Radiation Oncology & Therapy The First Hospital of Jilin University Changchun China; ^2^ Department of Radiation Oncology The First Hospital of Jilin University Changchun China; ^3^ NHC Key Laboratory of Radiobiology School of Public Health, Jilin University Changchun China

**Keywords:** high‐grade gliomas, immunohistochemical, overall survival, progression‐free survival, Trib3

## Abstract

**Objectives:**

High‐grade gliomas (HGGs) are highly malignant, aggressive, and have a high incidence and mortality rate. The aim of this study was to investigate survival outcomes and prognostic factors in patients with HGGs.

**Methods:**

In this retrospective study, a total of 159 patients with histologically confirmed HGGs were included. The recruitment period was from January 2011 to December 2019. We evaluated patient demographic data, tumor characteristics, treatment methods, immunocytochemistry results, overall survival (OS) time, and progression‐free survival (PFS) time using Kaplan–<>Meier survival analysis with log‐rank testing. Additionally, we employed Cox regression analysis to identify independent factors associated with survival outcomes.

**Results:**

Kaplan–Meier survival analysis revealed that the 1‐, 2‐, and 5‐years OS rates were 81.8%, 50.3%, and 12.6%, respectively. Similarly, the 1‐, 2‐, and 5‐years PFS rates were 50.9%, 22.4%, and 3.1%, respectively. The median OS duration was 35.0 months. The univariate analysis indicated that postoperative pathological classification, grade, and age were significantly associated with patient outcomes (*p* < 0.01). Among the patients, 147 received concurrent chemoradiotherapy, while 12 did not. The immunohistochemical markers of ki‐67, MGMT, IDH1R132H, and p53 demonstrated statistically significant differences in their prognostic impact (*p* = 0.001, *p* = 0.020, *p* = 0.003, and *p* = 0.021, respectively). In conclusion, we found that grades, age, pathological classification, ki‐67, MGMT, and IDH1R132H expression were statistically significantly associated with PFS (*p* < 0.01, *p* = 0.004, *p* = 0.003, *p* = 0.001, *p* = 0.036, and *p* = 0.028). Additionally, immunohistochemical expressions of TRIB3 and AURKA were significantly higher in patients with shorter survival (*p* = 0.015 and *p* = 0.023).

**Conclusions:**

Tumor grade and the use of concurrent chemoradiotherapy after surgery were independent prognostic factors that significantly influenced patient survival. Additionally, tumor grade and MGMT expression were found to be independent factors affecting progression‐free survival (PFS). Notably, the expression of TRIB3 and AURKA was higher in patients with poor survival outcomes.

## Graphical abstract

Tumor grade and concurrent chemoradiotherapy after surgery were independent prognostic factors affecting patients’ survival. Different grade, MGMT expression were also independent factors affecting PFS. The expression of TRIB3 and AURKA was increased in poor survival group.

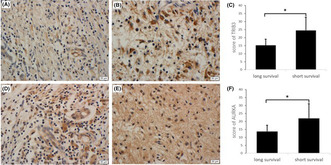

## 1 BACKGROUND

Gliomas, which derive from the progenitor cells or neuroglial stem, are responsible for the majority of deaths from primary brain tumors.^1^ The World Health Organization (WHO) classification of gliomas places gliomas into grades 1–4, with grades 1 and 2 indicating low‐grade gliomas and grades 3 and 4 high‐grade gliomas (HGGs). HGGs are highly malignant and invasive, with high morbidity and mortality. Surgical resection is the conventional treatment for HGGs, but it is difficult to completely remove clean because the boundary between normal tissue and tumor cells is not clear and tumor cells are highly infiltrative and invasive.^2^ Therefore, it is necessary to treat HGGs with adjuvant postoperative chemoradiotherapy as soon as possible. Current NCCN guidelines propose that surgical resection, concurrent chemoradiotherapy, and adjuvant temozolomide (TMZ) as a standard treatment for HGGs.^3^

Classic research has shown that radiotherapy in combination with chemotherapy, followed by up to six cycles of adjuvant TMZ, significantly boosted 5‐year overall survival (OS) and progression‐free survival (PFS) compared to radiotherapy alone.^4^ In addition, Karnofsky performance status (KPS) score, age, tumor volume, extent of surgical resection, and radiotherapy dose also affected the survival time of patients.^5^, ^6^, ^7^, ^8^ In addition to standard therapy, the research of targeted therapy, immunotherapy, and tumor‐treating fields therapy is also advancing. Some biological markers suggest that gliomas may have more drug options, such as IDH mutation, EGFR amplification, and BRAF V600E mutations. Ivosidenib, IDH1/2 inhibitors, had a higher median PFS of 13.6 months for recurrent IDH‐mutated high‐grade gliomas.^9^ The median survival time of anti‐EGFR antibody nimotuzumab combined with radiotherapy in the treatment of newly diagnosed HGGs was 17.8 months.^10^ For recurrent gliomas with BRAF V600E mutation, trametinib combined with dabraafenib can be used. More biomarker may herald a more targeted drugs, this is also helpful for the treatment of HGGs.

HGGs carry a dismal prognosis and exhibit a high recurrence rate, emphasizing the crucial importance of investigating prognostic factors that impact glioma survival. In this study, 159 patients with HGGs were selected and the association of relevant factors including biomarkers with prognosis was evaluated. The objective of this study is to ascertain the factors that influence the OS and PFS of patients with HGGs, thereby aiding in the exploration of advanced treatment strategies.

## 2 MATERIALS AND METHODS

### 2.1 Patients

This study retrospectively analyzed 159 HGGs patients treated in the first hospital of Jilin University from January 2011 to December 2019. All patients were newly diagnosed as grade III–IV (WHO) glioma, and excluded if they were pregnant or lactating women, or had other brain tumors. The ethics committee of the First Hospital of Jilin University has reviewed and approved this study, with the ethical number of 2022‐KS‐010.

### 2.2 Data collection

Clinical data were retrieved from the medical records of HGGs patients for statistical analysis. The data included HGGs patient's age, grade, histology, gender, KPS, tumor location, tumor lesion number, tumor diameter, treatment approaches (surgery, radiotherapy, and chemotherapy), immunohistochemistry (IHC) expression (ki‐67, O6‐methylguanine‐DNA methyltransferase (MGMT), isocitrate dehydrogenase 1 gene (IDH1) R132H, epidermal growth factor receptor (EGFR), and P53), the OS, and PFS time.

### 2.3 Immunochemical staining and analyses

Among 159 patients, 20 glioblastoma patients with the longest survival periods and 20 glioblastoma patients with the shortest survival periods were selected, and those with unresected surgery and without concurrent chemoradiotherapy and at least four cycles of chemotherapy were excluded. The postoperative pathological tissues of this 40 patients were stained by IHC staining of Tribbles 3 (TRIB3) and Aurora kinase A (AURKA). IHC staining was performed as previously reported. Primary antibodies included TRIB3 (rabbit monoclonal, 1:100 dilution, #ab75846, Abcam, Cambridge, UK) and AURKA (rabbit polyclonal, 1:100 dilution, #ab52974, Abcam, Cambridge, UK). The secondary antibody was purchased from SeraCare (goat anti‐rabbit, #5220–0336, SeraCare, Massachusetts, USA). The formula number of pixels in a zone multiplied by score of the zone divided by total number of pixels in the zone was used to calculate the IHC scores of each patient by ImageJ software, and the scores were statistically analyzed.^11^

### 2.4 Therapy

All patients received standard treatment, including maximum tumor resection, postoperative concomitant chemoradiotherapy, and sequential chemotherapy. Radiotherapy modality was generally chosen as intensity‐modulated radiation therapy (IMRT) or VMAT. The target area was outlined according to preoperative and postoperative CT and MRI. The median radiation dose was 60 Gy (12–66 Gy). The chemotherapy regimen was usually oral TMZ during radiotherapy, and took it 4 weeks after radiotherapy, generally lasting for more than half a year.

### 2.5 Statistical analysis

SPSS 21.0 (SPSS Inc., Chicago IL, USA) was used for statistical analysis, and the qualitative data were expressed using rate. OS was estimated from the date of diagnosis to the date of death. PFS was estimated from diagnosis to disease progression or death or the date of last follow‐up. Kaplan–Meier method were used to estimate OS and PFS among different factors and log‐rank method was used to test the differences in survival time distributions. The statistically different factors observed in the log rank method were incorporated into the Cox regression model using the forward: LR method to evaluate the independent risk factors affecting the prognosis of HGGs. The statistical significance of differences was set at *P* < 0.05.

## 3 RESULTS

### 3.1 Patients' baseline characteristics

Their characteristics of 159 patients are presented in the Table 1. There were 94 males (59.1%) with median age of 49.5 years, and 65 females (40.9%) with median age of 49 years. These HGGs patients were divided into 53 grade III and 106 grade IV, consisting of 95 cases of GBM, 19 cases of anaplastic oligodendroglioma, 11 cases of anaplastic astrocytoma, 16 cases of oligoastrocytoma, 5 cases of oligodendroglioma, and 13 cases of astrocytoma. Almost half of the patients had a preoperative KPS more than 60 (*n* = 97, 61.0%). Most patients underwent total resection, and only 4 patients did not undergo surgery and 11 patients underwent subtotal surgery. Most patients received postoperative irradiation, only 12 patients refused radiotherapy at the initial diagnosis of HGGs because of physical intolerance or personal reasons. 15.1% of patients received radiation doses no more than 54 Gy, 48.4% of patients received radiation doses between 54 and 60 Gy, and 36.5% of patients received doses more than 60 Gy. Among these patients, 44 patients were given radiotherapy less than 4 weeks after operation, while most radiotherapy (*n* = 100, 62.9%) started more than 4 weeks after section. Tumor location was as followed: right hemisphere (*n* = 70, 44.0%), left hemisphere (*n* = 81, 50.9%) and other sites (*n* = 8, 5.0%). Around 63.5% of the tumors were single lesion, and the remainder were diffuse multiple lesions. Expressions of ki‐67, IDH1 R132H, MGMT, p53, and EGFR were detected by IHC, and the results are reported in Table 1.

**TABLE 1:** Clinical characteristics of 159 high‐grade gliomas.

Variable	No.	%
Age		
<40 years	40	25.16
≥40 years	119	74.84
Grade		
III	53	32.33
IV	106	66.67
Histology		
Glioblastoma(GBM)	95	59.75
Anaplastic oligodendroglioma(AO)	19	11.95
Anaplastic astrocytoma(AA)	11	6.92
Oligoastrocytomas(OA)	16	10.06
Oligodendroglioma(O)	5	3.14
Astrocytoma(A)	13	8.18
Gender		
Male	94	59.12
Female	65	40.88
KPS		
>60	97	61.01
≤60	62	38.99
Location		
Right hemisphere	70	44.03
Left hemisphere	81	50.94
Others	8	5.03
Lesion number		
Single	101	63.52
Multiple	58	36.48
Diameter		
<4 cm	38	23.90
≥4 cm	95	59.75
Missing	26	16.35
Radiotherapy		
Yes	147	91.82
No	12	8.18
Concurrent chemoradiotherapy		
Yes	144	90.57
No	15	9.43
Interval between radiotherapy and surgery		
<4 weeks	44	27.67
≥4 weeks	100	62.89
Missing	15	9.43
Radiation dose		
≤54 Gy	24	15.09
54–60 Gy	77	48.43
≥60 Gy	58	36.48
Ki‐67		
<15%	35	22.01
≥15%	96	60.38
Unknown	28	17.61
MGMT		
Low	42	26.41
High	70	44.03
Unknown	47	29.56
IDH1R132H		
Negative	79	49.69
Positive	26	16.35
Unknown	54	33.96
P53		
Negative	28	17.61
Positive	98	61.64
Unknown	33	20.75
EGFR		
Negative	12	7.55
Positive	43	27.04
Unknown	104	65.41
Surgical treatment		
No operation	4	2.52
Subtotal resection	11	6.92
Total resection	144	90.57

### 3.2 Length of OS time and PFS time

The median OS time for the whole group was 35.0 months (95% CI 28.0–42.0 months), and the mean OS time was 52.7 months (95% CI 44.9–60.5 months). The OS rate of 6 months, 1, 2, 3, and 5 years were 96.9%, 81.8%, 50.3%, 31.4%, and 12.6%, respectively. The median PFS time of patients was 13.0 months (95% CI 10.4–15.64 months). The PFS of 6 months, 1, 2, 3, and 5 years were 78.6%, 50.9%, 22.4%, 8.81%, and 3.14%, respectively.

### 3.3 Predictors of survival in univariate analysis

**FIGURE 1:**
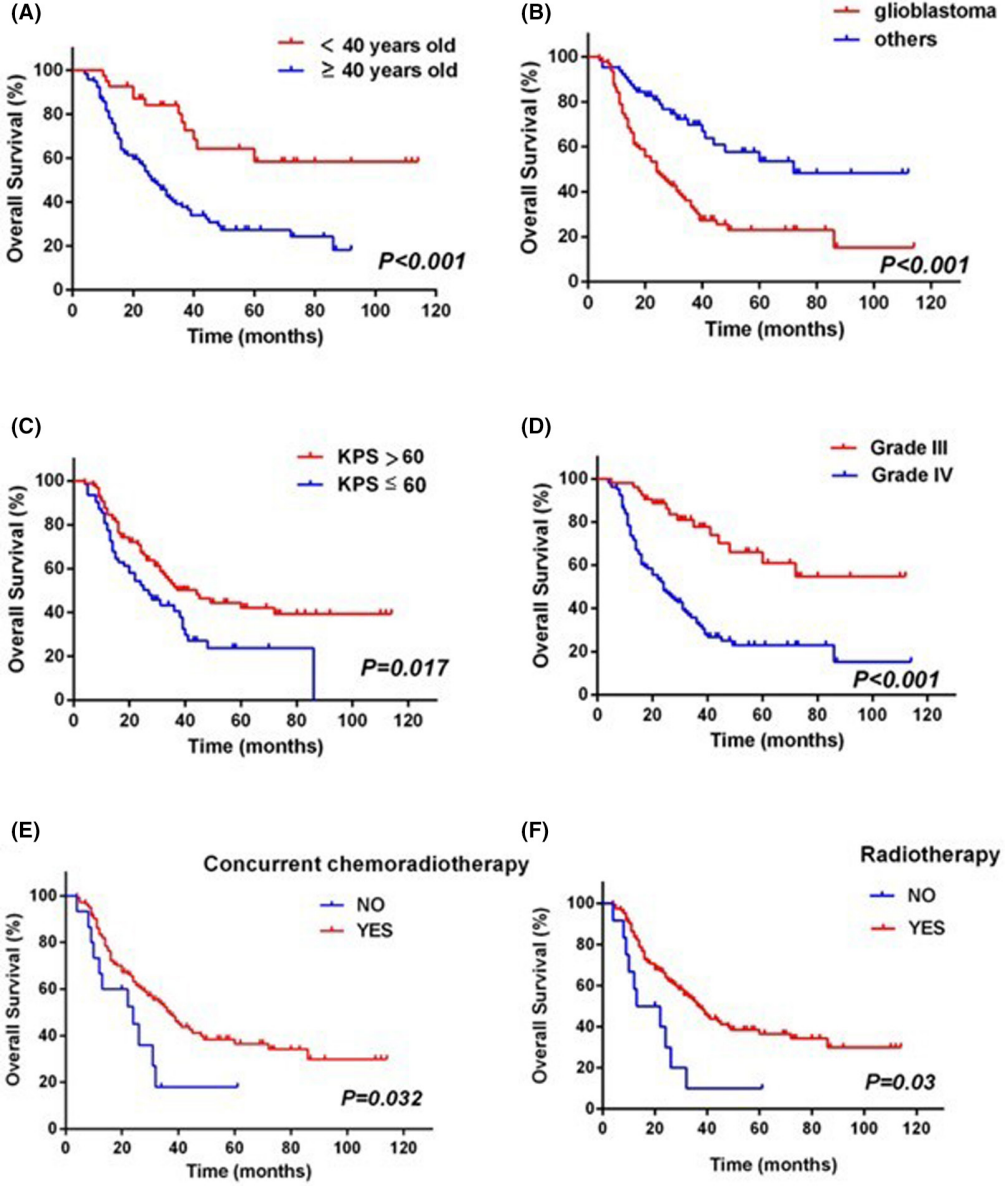
Kaplan–Meier analysis showed that age of onset, tumor type, KPS score, WHO grade, postoperative concurrent chemoradiotherapy, and postoperative radiotherapy were significantly associated with overall survival in high‐grade glioma patients.

**FIGURE 2:**
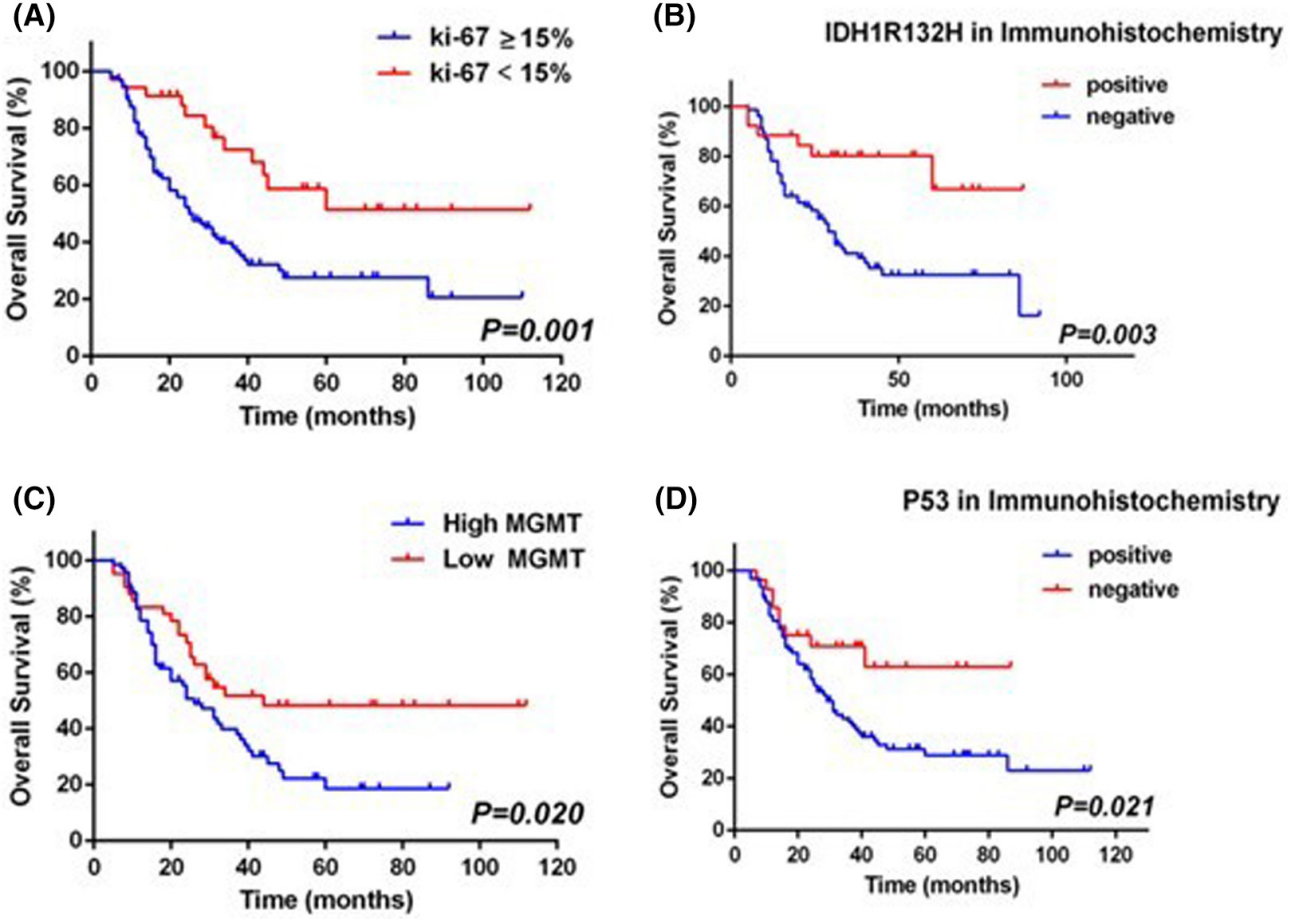
Kaplan–Meier analysis showed that ki67, IDH1R132, MGMT, and P53 were significantly associated with overall survival in high‐grade glioma patients.

The results of univariate analysis in OS are displayed in Table 2 and Figures 1, 2. In univariate analysis, age (*P* < 0.001), grade (*P* < 0.001), histology (*P* < 0.001), preoperative KPS (*P* = 0.008), radiotherapy (*P* = 0.003), concurrent chemoradiotherapy (*P* = 0.032), and some clinicopathological parameters composing of ki‐67 (*P* = 0.001), MGMT (P = 0.020), IDH1 R132H (*P* = 0.003), and P53 (*P* = 0.021) were statistically significant prognostic factors of OS. Somewhat surprisingly, the tumor diameter (*P* = 0.527), the interval between radiotherapy and surgery (*P* = 0.074), radiation dose (*P* = 0.074), and surgical type (*P* = 0.285) were not associated with OS benefit in HGGs patients.

**TABLE 2:** Variables related to overall survival in univariate analysis and mulvariate Cox analysis.

Variable	Log‐rank test (*P* value)	Median OS (months)	Hazard ratio (95% confidence interval)	Cox regression analysis (*P* value)
Age				
<40 years	*P* < 0.001	N/A		
≥40 years		26		
Grade				
III	*P* < 0.001	N/A	2.553 (1.182–5.514)	*P* = 0.017
IV		24		
Histology				
Glioblastoma	*P* < 0.001	24		
Others		72		
KPS				
>60	*P* = 0.008	44		
≤60		27		
Gender				
Male	*P* = 0.456	32		
Female		37		
Location				
Right hemisphere	*P* = 0.792	33		
Left hemisphere		34		
Others		41		
Lesion number				
Single	*P* = 0.378	31		
Multiple		49		
Diameter				
<4 cm	*P* = 0.527	29		
≥4 cm		33		
Radiotherapy				
Yes	*P* = 0.003	37	3.539 (1.351–9.273)	*P* = 0.010
No		13		
Concurrent chemoradiotherapy				
Yes	*P* = 0.032	37		
No		24		
Interval between radiotherapy and surgery				
<4 weeks	*P* = 0.074	31		
≥4 weeks		41		
Radiation dose				
≤54 Gy	*P* = 0.823	31		
54–60 Gy		32		
≥60 Gy		36		
Ki‐67				
<15%	*P* = 0.001	N/A		
≥15%		26		
MGMT				
Low(<10%)	*P* = 0.020	44		
High(≥10%)		26		
IDH1R132H				
Negative	*P* = 0.003	29		
Positive		N/A		
P53				
Negative	*P* = 0.021	N/A		
Positive		29		
EGFR				
Negative	*P* = 0.194	31		
Positive		20		
Surgical treatment				
No operation	*P* = 0.285	13		
Subtotal resection		25		
Total resection		36		

**FIGURE 3:**
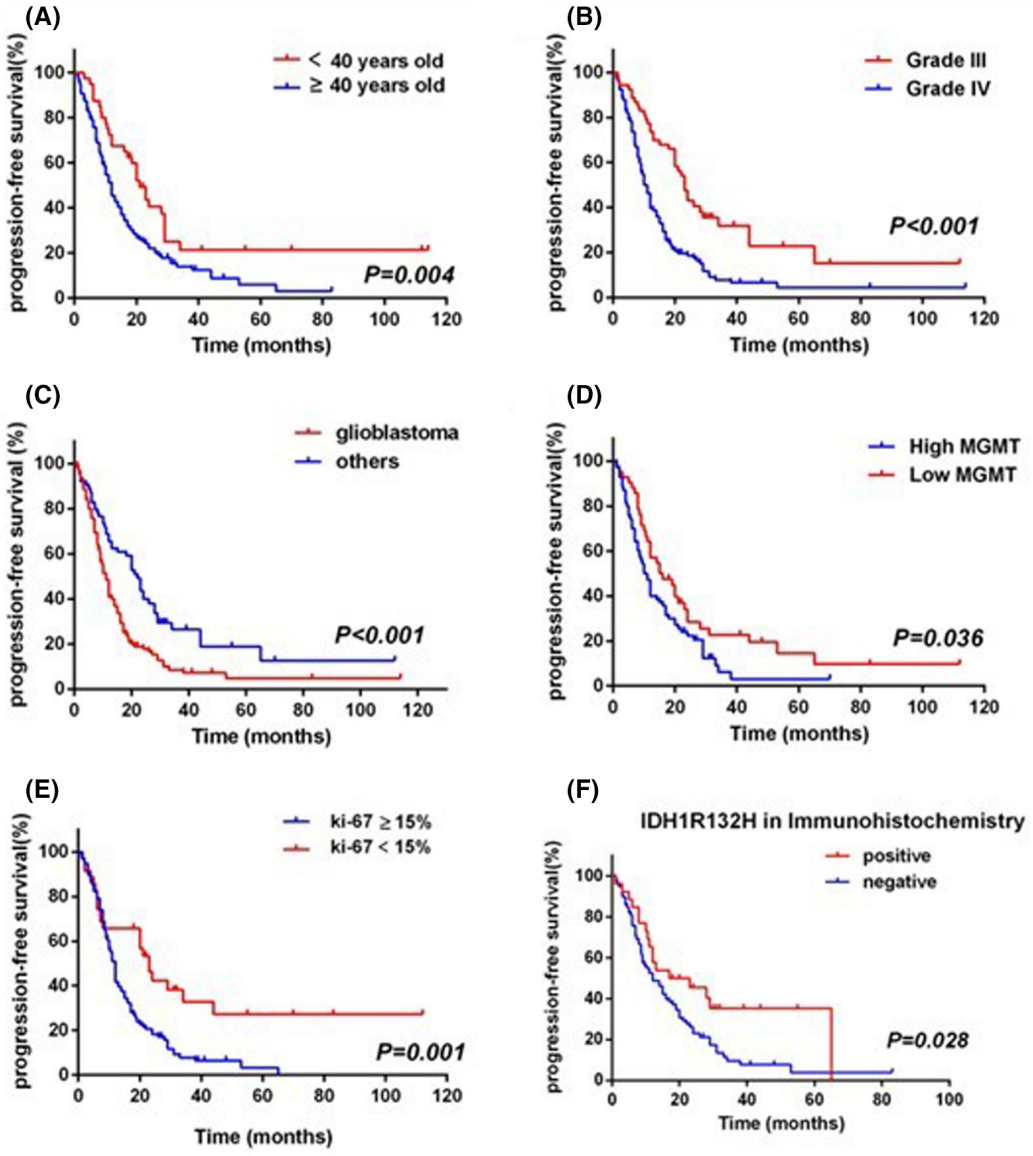
Kaplan–Meier analysis showed that age, tumor grade, tumor type, MGMT, Ki‐67, and IDH1R132 were significantly associated with progression‐free survival in high‐grade glioma patients.

The results of univariate analysis in PFS are displayed in Table 3 and Figure 3. We found that statistically significant prognostic factor of PFS included age (*P* = 0.004), grade (*P* < 0.001), histology (*P* < 0.001), immunohistochemistry of ki‐67 expression (*P* = 0.001), MGMT expression (*P* = 0.036), and IDH1 R132H expression (*P* = 0.028).

**TABLE 3:** Variables related to progress‐free survival in univariate analysis and mulvariate Cox analysis.

Variable	Progression(*n*)	Log‐rank test (*P* value)	Median PFS (months)	Cox regression analysis (*P* value)	Hazard ratio (95% confidence interval)
Age					
<40 years	29	*P* = 0.004	21		
≥40 years	104		12		
Grade					
III	36	*P* < 0.001	23	*P* = 0.007	2.211 (1.242–3.937)
IV	97		10		
Histology					
Glioblastoma	87	*P* < 0.001	11		
Others	46		22		
KPS					
>60	81	*P* = 0.135	15		
≤60	52		12		
Gender					
Male	80	*P* = 0.829	12		
Female	53		14		
Location					
Right hemisphere	61	*P* = 0.259	12		
Left hemisphere	63		15		
Others	9		10		
Lesion number					
Single	88	*P* = 0.539	14		
Multiple	45		12		
Diameter					
<4 cm	30	*P* = 0.981	11		
≥4 cm	82		12		
Radiotherapy					
Yes	122	*P* = 0.071	14		
No	11		8		
Concurrent chemoradiotherapy					
Yes	121	*P* = 0.465	13		
No	12		10		
Interval between radiotherapy and surgery					
≤4 weeks	36	*P* = 0.633	14		
>4 weeks	83		13		
Radiation dose					
≤54 Gy	16	*P* = 0.167	13		
54–60 Gy	62		13		
≥60 Gy	55		12		
Ki‐67					
<15%	22	*P* = 0.001	23		
≥15%	89		12		
MGMT					
Low	34	*P* = 0.036	15	*P* = 0.034	1.737 (1.043–2.893)
High	62		10		
IDH1R132H					
Negative	68	*P* = 0.028	12		
Positive	17		17		
EGFR					
Negative	10	*P* = 0.467	18		
Positive	38		10		
P53					
Negative	20	*P* = 0.070	13		
Positive	86		12		
Surgical treatment					
No operation	4	*P* = 0.743	6		
Subtotal resection	10		16		
Total resection	119		13		

Immunohistochemical results showed that high expression of ki67, low expression of MGMT, negative IDH, and positive TP53 were significantly correlated with longer OS and PFS.

### 3.4 Prognostic factors in multivariate analysis

The results of multivariate analysis in OS are displayed in Table 2. The multivariate survival analysis identified the following parameters as independent factors associated with prolonged OS time: the grade of gliomas (*P* = 0.017, HR 2.553, 95%CI 1.2–5.5) and patients received radiotherapy (*P* = 0.010, HR 3.539, 95%CI 1.4–9.3).

The results of multivariate analysis in PFS are displayed in Table 3. The grade of gliomas (P = 0.034, HR 1.737, 95%CI 1.0–2.93) and MGMT expression in immunohistochemistry (*P* = 0.007, HR 2.211, 95%CI 1.2–3.9) were the independent prognostic factors of PFS.

**FIGURE 4:**
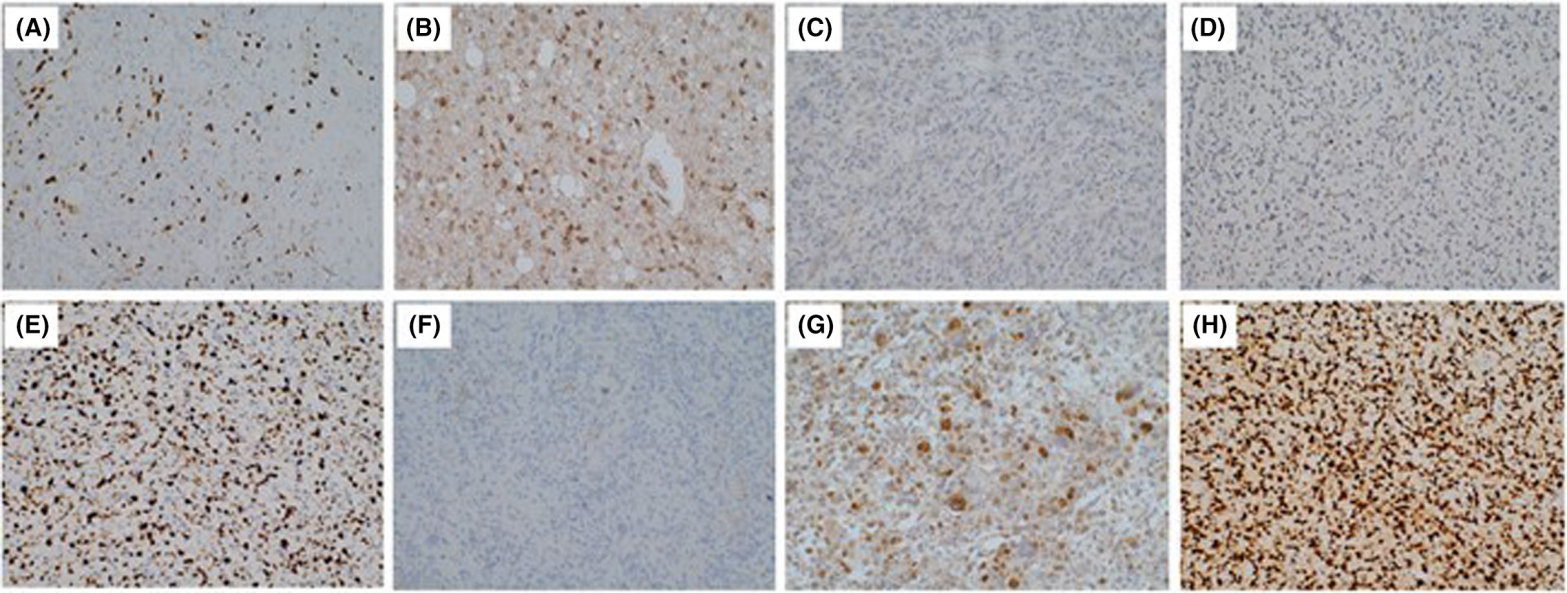
(A–D) The immunohistochemical expression of ki67, IDH, MGMT, and P53 in patients with long survival. (E–H) The immunohistochemical expression of ki67, IDH, MGMT, and P53 in patients with short survival.

Our analysis reveals that patients with pathological of grade IV and those who did not receive postoperative radiotherapy are at higher risk of mortality. More noteworthy, high expression of MGMT in immunohistochemistry and GBM were correlation to tumor progression (Figure 4A–H).

### 3.5 High expression of TRIB3 and AURKA was associated with poor survival

**FIGURE 5:**
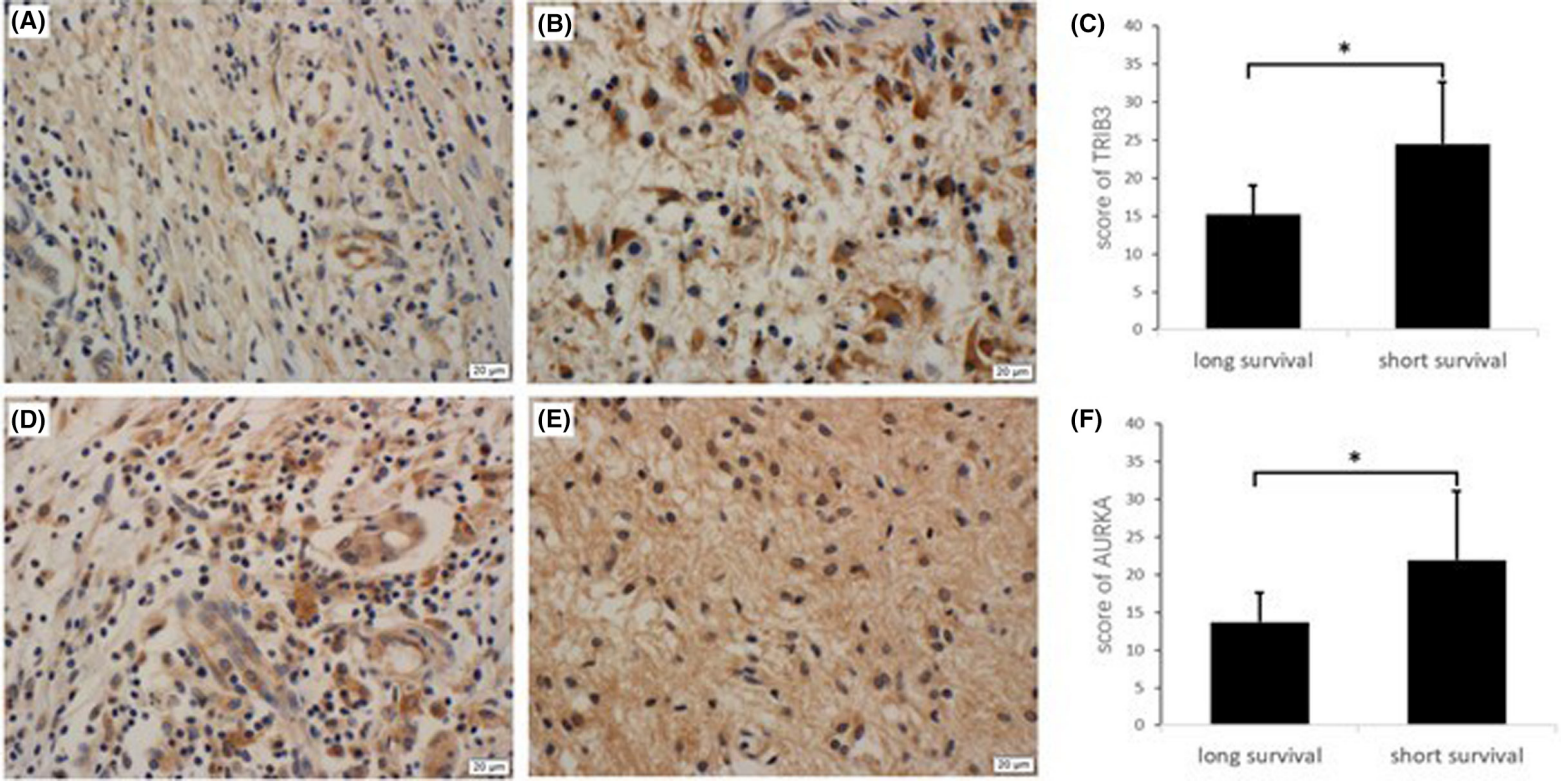
(A, B) Immunochemical staining indicated that the expression of TRIB3 was significantly higher in patients with short survival than in patients with long survival. (C) The TRIB3 immunohistochemical score of the long survival group was significantly higher than that of the short survival group. (D, E) Immunochemical staining indicated that the expression of AURKA was significantly higher in patients with short survival than in patients with long survival. (F) The AURKA immunohistochemical score of the long survival group was significantly higher than that of the short survival group. *P < 0.05.

IHC staining showed that the expression of TRIB3 and AURKA was significantly increased in patients with short survival (Figure 5A,B,D,E). The short survival group had a significantly higher TRIB3 score than the long survival group (24.5 vs. 15.2, *P* = 0.015), and the AURKA also exhibits a similar phenomenon (21.9 vs. 13.7, *P* = 0.023), respectively (Figure 5C,F).

## 4 DISCUSSION

HGGs are among the most malignant tumors with high aggressiveness and heterogeneity. The prognostic factors and treatment of HGGs are complex and changeable. Therefore, the formulation of individualized treatment plan according to the prognostic factors can guide clinical work and improve the treatment effect. Surgical resection is the most important treatment for glioma, and the maximum resection of the tumor may improve the survival of patients. A meta‐analysis showed significantly lower mortality with GTR compared with STR. Any resection, as compared with biopsy alone, similarly reduced the risk of death at 1 and 2 years.^12^ Large the scope of extended resection is associated with more survival benefit for HGGs. A study revealed that independent predictors of postoperative epilepsy included temporal lobe involvement, preoperative glioma‐related epilepsy, lower WHO grade and non‐gross‐total resection.^13^ For recurrent HGGs, re‐resection or not had no significant effect on median OS.^14^ Although our results suggested that the surgical method did not provide a statistically significant prolongation survival time. This may be attributed to the limited number of patients with partial resection and simple biopsy, and the statistician may have data bias. Although no statistical differences were observed, the median OS without surgery, partial resection, and total resection were 13.0 months, 25.0 months, and 36.0 months, respectively. It can be seen that total resection still has a better prognosis compared with the other two groups.

An epidemiological statistic revealed that men were more prone to gliomas than women.^15^ Our study did not indicate there have significant difference in OS and PFS between males and females. However, both median OS (37.0 months vs. 32.0 months) and median PFS (14.0 months vs. 12.0 months) were higher in females than males. Oligodendroglioma occurred predominantly in the median age of 43 years old, diffuse astrocytoma induced at the median age of around 48 years old, anaplastic oligodendroglioma developed at the median age of 49, anaplastic astrocytoma occurred at around 53 years old, whereas GBM peak in the median age of 64.^16^ We revealed that the median ages of oligodendroglioma, astrocytoma, anaplastic oligodendroglioma, anaplastic astrocytoma, and GBM were 46.0, 44.0, 42.0, 45.0, and 55.5 years old. These results suggested that HGGs mainly occurred in 40–60 years old. Our findings revealed that patients younger than 40 years old had significantly better OS and PFS than patients in the other ages(*P* < 0.001 and *P* = 0.004).

Radiotherapy is an essential therapeutic method for HGGs patients. A clinical trial found no statistically significant difference in survival rate in the dose range of 60–80 Gy^17^; however, another study found that patients received 80 Gy and 90G y had significantly longer OS than patients received 60 Gy.^18^ In our study, no significant difference in OS or PFS was found between radiotherapy doses, and patients who received doses more than 60 Gy had the highest median OS (36.0 months vs. 32.0 months) but the lowest median PFS (12.0 months vs. 13.0 months). It is difficult for conventional radiotherapy technology to carry out radical treatment for tumors close to dangerous organs. Patients receive particles, such as protons or carbon ions, and this modality with higher linear energy transfer can improve the dose distribution, induces DNA double strand breaks, killing cell more efficiency, which can ameliorate the curative effect of radiotherapy. The utilization of radiation modalities that promote enhanced local dose deposition, escalate irradiation dosage for the biological target volume, and enable intraoperative radiotherapy may represent the prospective developmental trajectory for high‐grade glioma radiotherapy in the future. In our study, we use are x‐ray radiation, compared with no postoperative radiotherapy, postoperative conventional radiotherapy can improve the median OS of HGGs patients significantly, which is consistent with the conclusion of our study (*P* = 0.003).

IDH1 mutation at codon 132 is related to the prognosis of HGGs. Patients with IDH mutant glioblastoma usually have a better prognosis than patients with IDH wild‐type glioblastoma. Ivosidenib had a higher median PFS of 13.6 months for recurrent IDH‐mutated high‐grade gliomas.^10^ However, the CNS WHO 5 has classified IDH‐mutant gliomas into oligodendroglioma and astrocytoma, and glioblastomas are all IDH wild type, so glioblastomas cannot benefit from ivosidenib, but for oligodendroglioma and astrocytoma, this is undoubtedly a breakthrough in the treatment.

MGMT promoter methylation is a biomarker to predict the benefit from TMZ chemotherapy. The gold standard examination for MGMT methylation is polymerase chain reaction (PCR), but for some cases where the examination conditions are limited or PCR cannot be performed due to personal reasons, it can also be replaced by immunohistochemistry (IHC). Anda et al.^19^ found that the median PFS was 9.0 months and the median OS was 12.0 months in patients of high MGMT expression, while the median PFS was 15.0 months and the median OS was 22.0 months in patients of low MGMT expression. High MGMT expression is associated with poor prognosis. This is consistent with our research conclusions. Among the enrolled patients, 70 patients had high MGMT expression and 42 patients had low MGMT expression, except for patients with unknown MGMT expression. The median OS of low and high MGMT expression were 44.0 and 26.0 months, respectively (*P* = 0.020), median PFS were 15.0 and 10.0 months, respectively (*P* = 0.036).

P53 is a tumor‐suppressor gene involved in the occurrence of HGGs. As a highly specific marker, P53 is highly positive in HGGs, and the positive rate is higher than that of patients with low‐grade gliomas. Overexpression of P53 suggests that the prognosis of patients is poor. In our study, p53‐positive indicated poor prognosis. Compared with negative, there was a significant difference in median OS (*P* = 0.021), but no significant difference in median PFS (*P* = 0.070). This result was consistent with a retrospective analysis of anaplastic gliomas.^20^

Ki‐67 is a nuclear protein that describes the proliferative stage of the cell cycle and reflects the proliferative activity of HGGs cells. It is an independent prognostic factor in gliomas, and a higher expression content is associated with a worse prognosis.^21^ In our study, the median PFS of patients with ki‐67 ≥ 15 and those with ki‐67 < 15 were 12.0 and 23.0 months, respectively (*P* = 0.001). The median OS of patients with ki‐67 ≥ 15 was 26.0 months, while those with Ki‐67 < 15 had not yet reached (*P* = 0.001).

We examined several biomarkers that are not routinely used in glioma IHC detection and found that TRIB3 and AURKA were associated with poor prognosis of the disease. Multiple studies have shown that TRIB3 expression is upregulated in response to various stressors, including oxidative stress, metabolic stress, as well as endoplasmic reticulum (ER) stress.^22^ Recently, several studies have identified TRIB3 as a crucial regulator in tumorigenesis and tumor progression. TRIB3 is overexpressed in various cancer tissues and is closely associated with poor prognosis in patients, including breast cancer,^23^ and colorectal cancer,^24^ lung cancer.^25^ Unfortunately, the precise roles of TRIB3 in high‐grade gliomas (HGGs) remain elusive. The mRNA expression of TRIB3 in glioblastoma clinical samples was higher than that in normal brain tissue.^26^ TRIB3 is also expected to be a new target for glioma treatment. In our study, we selected 40 patients with glioblastoma according to different prognosis. Through IHC analysis of these patients, we found the expression of TRIB3 in the short‐term survival was significantly higher than those in the long‐term survival. This difference was verified by ImageJ software analysis (*P* = 0.015).

AURKA is a serine/threonine kinase that regulates cellular mitosis and is located on chromosome 20q13.2.^27^ As a cell cycle regulator, AURKA overexpression leads to centrosome expansion and aneuploidy. AURKA promotes tumorigenesis by participating in epithelial‐mesenchymal transformation, proliferation and metastasis of cancer cells, apoptosis, and self‐renewal of stem cells.^28^ In glioma, inhibition of AURKA expression can inhibit the growth of tumor cells, which can be used as a potential therapeutic target. Similar to TRIB3, we also found that the expression of AURKA in the short survival was significantly higher (P = 0.023). It is anticipated that further investigation into TRIB3 and AURKA could lead to the development of novel therapeutic targets for glioma, such as MGMT and EGFR.

Reviewing our data, the overall OS and PFS were higher than the guidelines, analysis may be related to the following factors. Most patients received multidisciplinary consultation, and this mode of treatment can make the treatment of patients more standardized and timely. A subset of patients received longer cycles of TMZ chemotherapy. The longest surviving patient with glioblastoma was 86 months, and this patient received TMZ for a total of 80 months. Long cycles of temozolomide have also been reported to improve survival.^29^ Some patients who have survived for a long time have a pathological type of astrocytoma or oligodendroglioma, which has a better prognosis. Moreover, because of lack of the molecular pathology information in our samples, which may cause some statistical bias, such as the prognosis of grade IV patient with IDH mutated is significantly better than that of grade IV patients with IDH wild type, two different subtypes of gliomas may have different prognostic factors affecting OS and PFS, which is an underexplored part of our study.

In conclusion, this is a meaningful retrospective study with considerable number of HGGs patients, and we did a full‐scale analysis. Age, the grade and histology of tumor, preoperative KPS score, radiotherapy, concurrent chemotherapy, the expression of p53, MGMT, IDH1 R132H, and ki‐67 correlate significantly with the survival prognosis of these patients with HGGs. The high expression of TRIB3 and AURKA immunohistochemistry may indicate a shorter survival time of patients. Through this study, we can provide evidence for judging HGGs patients prognosis and a direction for the exploration of HGGs treatment.

## AUTHOR CONTRIBUTIONS

**Weiyan Shi:** Data curation (equal); investigation (equal); methodology (equal); writing – original draft (equal). **Xuanzhong Wang:** Methodology (equal). **Shiyu Liu:** Formal analysis (equal); software (equal). **Zhuangzhuang Zheng:** Formal analysis (equal); software (equal). **Lihua Dong:** Conceptualization (equal); validation (equal); writing – review and editing (equal). **Xin Jiang:** Conceptualization (equal); funding acquisition (equal); project administration (equal); supervision (equal); validation (equal); visualization (equal); writing – review and editing (equal).

## FUNDING INFORMATION

This research was funded by the Jilin Provincial Science and Technology Foundations, grant number: 20230508064RC.

## CONFLICT OF INTEREST STATEMENT

The authors declare no conflict of interest.

### ETHICS STATEMENT

The procedures followed in this study were reviewed and approved by the Ethics Committee of the First Hospital of Jilin University, which authorized the collection of partial tissue samples from patients in our institution: 2022‐KS‐010.

Informed Consent: N/A.

Registry and the Registration No. of the study/trial: N/A.

Animal Studies: N/A.

## Data Availability

All data generated or analyzed during this study are included in this published article. The datasets used or analyzed during the current study are available from the corresponding author on reasonable request.
